# Degeneration of three or more lumbar discs significantly decreases lumbar spine/hip ROM ratio during position change from standing to sitting in AVN patients before THA

**DOI:** 10.1186/s12891-020-3043-9

**Published:** 2020-01-18

**Authors:** Jianming Gu, Huixiong Feng, Xiao Feng, Yixin Zhou

**Affiliations:** Department of Orthopedics, Beijing Jishuitan Hospital, Fourth Clinical College of Peking University, Beijing, 100035 China

**Keywords:** Avascular necrosis; hip–spine syndrome, Sagittal balance, Total hip arthroplasty

## Abstract

**Background:**

Limitations in the lumbar spine movement reduce lumbar vertebral motion and affect spinopelvic kinematics. We studied the influence of lumbar intervertebral disc degeneration on spinofemoral movement, from standing to sitting, in patients undergoing total hip arthroplasty (THA).

**Methods:**

Of 138 consecutive patients scheduled for THA due to unilateral avascular necrosis (AVN) of the femoral head, those with ≥3 discs with University of California at Los Angeles (UCLA) disc degeneration score > 1 were defined as the lumbar degenerative disc disease (LDD) group, and the remaining patients constituted d the control group. Full body anteroposterior and lateral EOS images in the standing and sitting positions were obtained. Pelvic incidence (PI), L1 slope (L1 s), lumbar lordosis angle (LL), pelvic tilt (PT), sacral slope (SS), femoral slope (Fs), sagittal vertical axis (SVA), hip flexion, lumbar spine flexion, and total spinofemoral flexion were measured on the images and compared between groups.

**Results:**

No significant between-group differences were observed in the height, weight, body mass index, AVN staging, or PI, SS, and Fs on standing. The LDD group included more females and older patients, had 5° lesser LL, 5° greater PT, and larger SVA. From standing to sitting, the PI remained constant in both groups. Total spinofemoral flexion was 7° less, lumbar spine flexion 16° less, L1 slope change 6° less, and SS change 8° less, and hip flexion was 7° more in the LDD than in the control group. The spine/hip flexion ratio was significantly lower in the LDD group (0.3 versus 0.7; *p* < 0.001). On regression analysis, the LDD group (p < 0.001) and older age (*p* = 0.048) but not sex, weight, or height were significant univariate predictors of decreased spine/hip ratio.

**Conclusions:**

Patients with LDD leant more forward and had a larger pelvis posterior tilt angle on standing and a decreased lumbar spine/hip flexion ratio, with more hip joint flexion, on sitting, to compensate for reduced lumbar spine flexion. Surgeons should be aware that elderly patients with multiple LDD have significantly different spinofemoral movements and increased risk of posterior dislocation post-THA. Preoperative patient identification, intraoperative surgical technique modification, and individualized rehabilitation protocols are necessary.

## Background

Accurate orientation of hip components is essential during total hip arthroplasty (THA) to avoid impingement and to maintain stability during daily activities. Different definitions are available in literature for safe zones for acetabular components. Kummer et al. (1999) reported that adequate ranges of cup inclination and anteversion were 35–45° and 0–10°, respectively [1]. The classic safe zones (40° ± 10° inclination and 15° ± 10° anteversion in reference to the anterior pelvic plane) advocated by Lewinneck in 1978 have been widely applied by surgeons [2]. Widmer et al. (2004) reported that the sum of cup anteversion and 0.7 times the stem anteversion should be 37.3° to achieve maximal and stable postoperative hip range of motion (ROM) [3]. However, Abdel et al. (2016) reported that in more than 60% of 206 cases of dislocation after THA, acetabular components in the “safe zone” were noted [4].

The functional angles of the acetabular component in THA patients may change according to the body posture [5–8]. Studies have reported a 22.3–28.7° posterior pelvic tilt change from standing to sitting [6–8]. Babisch et al. (2008) reported that for every 1° change in the pelvic tilt, the cup inclination and anteversion changed by approximately 0.3° and 0.8°, respectively [5]. This pelvic movement functions as a protective mechanism against posterior dislocation when sitting or squatting. However, the limitation in lumbar spine motion may reduce this dynamic protection.

In addition to lumbar spine fusion, multiple lumbar disc degeneration (LDD) decreases the lumbar spine movement. McGregor et al. (1997) reported that patients with a degenerative lumbar spine or disc prolapse have an approximately 10° decrease in flexion and extension compared to the age-matched controls [9]. Lumbar spine movement reduction results in greater risk of dislocation and revision after THA [10, 11]. Elucidation and measurement of the effects of lumbar disc degeneration on spinopelvic movement are, thus, essential when preparing patients for THA. Therefore, this study investigated the effects of multiple lumbar intervertebral disc degeneration on spinofemoral movement during position change from standing to sitting in patients scheduled for hip joint replacement.

## Methods

### Study participants

With the approval of our institutional review board, 138 consecutive patients with unilateral avascular necrosis of femoral head (AVNFH) scheduled for primary THA from June to November 2018 were included in this retrospective study. AVNFH was staged according to the criteria of Ficat [12]. Verbal informed consent was obtained from the participants or their guardians.

The exclusion criteria were as follows: (1) hip diseases other than AVNFH, such as ankylosing spondylitis, developmental dysplasia of the hip, or rheumatoid arthritis, (2) squatting limitation (torso and hip flexion angle < 90°), (3) previous hip surgery, including hip arthroplasty, osteotomy, or osteosynthesis, (4) presence of lumbar scoliosis with an L1–S1 Cobb angle > 10°, and (5) history of spine compression fracture, spondylolysis, or spinal fusion surgery.

### Image acquisition

**Fig. 1:**
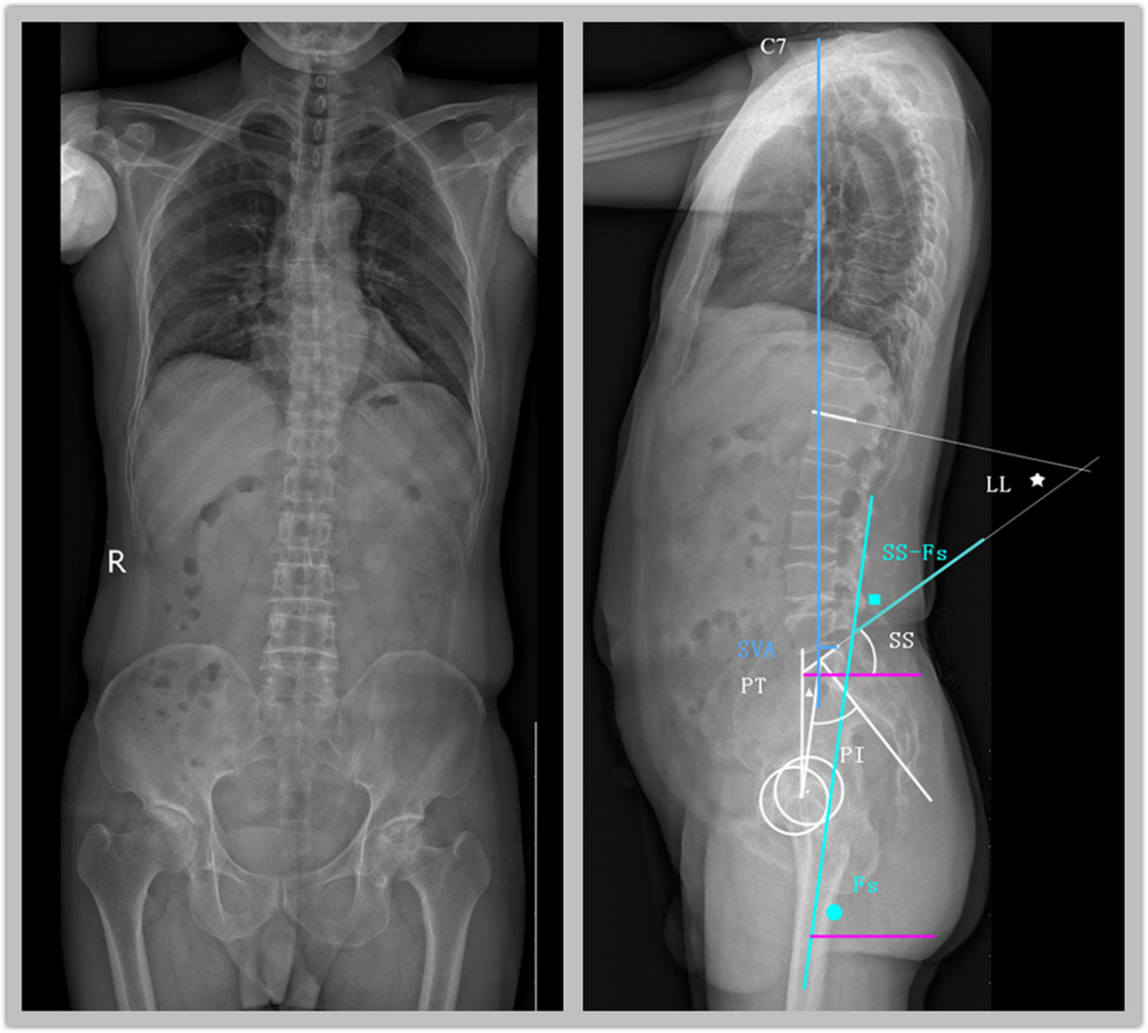
Anteroposterior and lateral EOS imaging and spinopelvic parameters. 1) Pelvic incidence (PI): the angle between the perpendicular plane to the middle of the S1 upper endplate and the line joining this point to the bicoxofemoral axis. 2) Sacral slope (SS): the angle between the horizontal plane and the upper endplate of S1. 3) Pelvic tilt (PT, ▲): the angle of the line connecting the middle S1 upper plate and bicoxofemoral axis to the vertical axis. 4) Lumbar lordosis angle (LL, ★): the angle between the superior endplates of L1 and S1. 5) Femoral slope (Fs, ○): the angle between the axis of the femur and horizontal plane. Hip flexion is expressed as the change in the angle (SS-Fs, □) between SS and Fs. 6) Sagittal vertical axis (SVA): the horizontal distance from the C7 plumb line to posterior superior corner of the superior margin of S1

All patients underwent biplanar full body examination using low radiation dose EOS imaging (EOS Imaging, Paris, France) in preparation for hip surgery [13]. Anteroposterior (AP) and lateral images in both standing and sitting positions (Fig. 1) were obtained and reconstructed using EOS Stereos® software [14]. Standing and sitting positions were standardized for all subjects. Patients were imaged with their femora aligned approximately parallel to the floor, seated at 90° of apparent hip flexion. The degrees of true flexion of the spine and hip were calculated using the imaging system. This relaxed seated position has been used in previous studies [15, 16].

Spine and pelvic sagittal parameters were measured using the EOS Stereos software and Surgimap software [17]. All measurements were performed by two trained researchers. In cases of greater than 10% mismatch between observers, another review was performed by a separate observer, and measurements were repeated and discussed till a consensus was reached.

### Parameter measurements

#### Group allocation

Lumbar degeneration severity was evaluated using the UCLA disc degeneration scale [18, 19]. Disk space narrowing, osteophyte formation, and endplate sclerosis were graded as 2, 3, and 4 points, respectively. Disks without these aforementioned changes were graded as 1 point. We defined the lumbar degenerative disc disease (LDD) group as patients with three or more discs graded > 1 point. Patients with fewer than three discs graded > 1 point were allocated to the control group.

#### Anatomic parameters

Pelvic incidence (PI) was defined as the angle between the perpendicular plane to the middle of the upper plate of S1 and the line joining this point to the bicoxofemoral axis.

#### Positional parameters

L1 slope (L1 s) was defined as the angle between the line along the L1 superior endplate and the reference horizontal line. The lumbar lordosis (LL) was measured from the superior endplate of L1 to the superior endplate of S1. Lordosis was expressed as a positive value and kyphosis as a negative value. The changes in LL during a position change from standing to sitting were calculated as lumbar spine ROM (lumbar spine flexion). (3) The sacral slope (SS) was defined as the angle between the horizontal plane and superior endplate of S1. The SS is a positional parameter that varies according to pelvis positioning: changes in SS indicate pelvic rotation. Posterior rotation (anterior superior iliac spine moving backwards) was recorded as a positive value, and anterior rotation was recorded as a negative value. Pelvic tilt (PT) was defined as the angle of the line connecting the middle S1 upper plate and the bicoxofemoral axis to the vertical axis. Femoral slope (Fs) was defined as the angle between the axis of the proximal femur and the horizontal plane, with changes in Fs indicating changes in femur position. Hip flexion was defined as movement between the femur and acetabulum of the pelvis, and was evaluated as the change in the angle between the superior S1 endplate and the proximal femur axis (SS-Fs). The total flexion of the hip–spine complex was defined as the change in the angle between the L1 superior endplate and the proximal femur axis, and comprised lumbar spine flexion (change in LL) and hip flexion (change in SS-Fs). The spine/hip flexion ratio was calculated as lumbar spine flexion divided by hip flexion. The sagittal vertical axis (SVA) was defined as the horizontal distance from the C7 plumb line to the posterior superior corner of the superior margin of S1 (Fig. 1).

### Data analysis

The normality of data distribution was determined using the Kolmogorov–Smirnov test. Radiographic parameters were compared using the Student’s *t*-test for continuous data and Fisher’s exact test for binominal data. Regression analyses were conducted to identify relationships between the spine/hip ratio and other variables. Data have been expressed as mean ± standard deviation. All data were analyzed with SPSS (version 21.0; IBM SPSS, Chicago, IL, USA) software. A *P*-value of < 0.05 was considered statistically significant.

## Results

A total of 138 cases of AVN were enrolled in this study (control group, *n* = 84; LDD group, *n* = 54). Overall, the mean age of the patients was 55.4 ± 12.9 years, and the mean body mass index (BMI) was 26.0 ± 9.2 kg/m^2^. There were more female (52.9%) than male patients, and the LDD group included significantly more females than the control group (*p* = 0.025). Most patients had Ficat Stage III AVN, and the AVN stage was comparable between the groups (*p* = 0.557). There were no significant differences in height, weight, or BMI between the groups. The demographic characteristics of the groups are shown in Table 1.

**Table 1:** Demographic data of the patients

Parameters	Control (*n* = 84) Mean ± SD	LDD (*n* = 54) Mean ± SD	*p* value	Total (*n* = 138) Mean ± SD
Female, number(%)	38 (45.2%)	35 (64.8%)	**0.02**	73(52.9%)
Age (years)	50.4 ± 12.6	63.2 ± 9.1	**< 0.01**	55.4 ± 12.9
Left hip (%)	38(45.2%)	25(46.3%)	0.90	63(45.7%)
Ficat stage III, number (%)	79(94.0%),	52 (96.3%)	0.55	131(94.9%)
Height (cm)	164.1 ± 11.9	163.1 ± 8.2	0.58	163.7 ± 10.6
Weight (kg)	69.3 ± 10.7	67.6 ± 10.2	0.34	68.7 ± 10.5
BMI (kg/m^2^)	26.5 ± 11.6	25.3 ± 3.0	0.46	26.0 ± 9.2

*BMI* Body mass index, *SD* Standard deviation

On standing, no significant differences were identified in PI, SS, or Fs between THE two groups. The LDD group had 5° lesser LL and a 2° smaller L1 slope than the control group. The PT was 5° greater in the LDD than in the control group. The SVA in the LDD group was 29 mm greater, indicating that the center of gravity was more anterior to the femoral head and the upper bodies of these patients leant more forward than those in the control group.

**Fig. 2:**
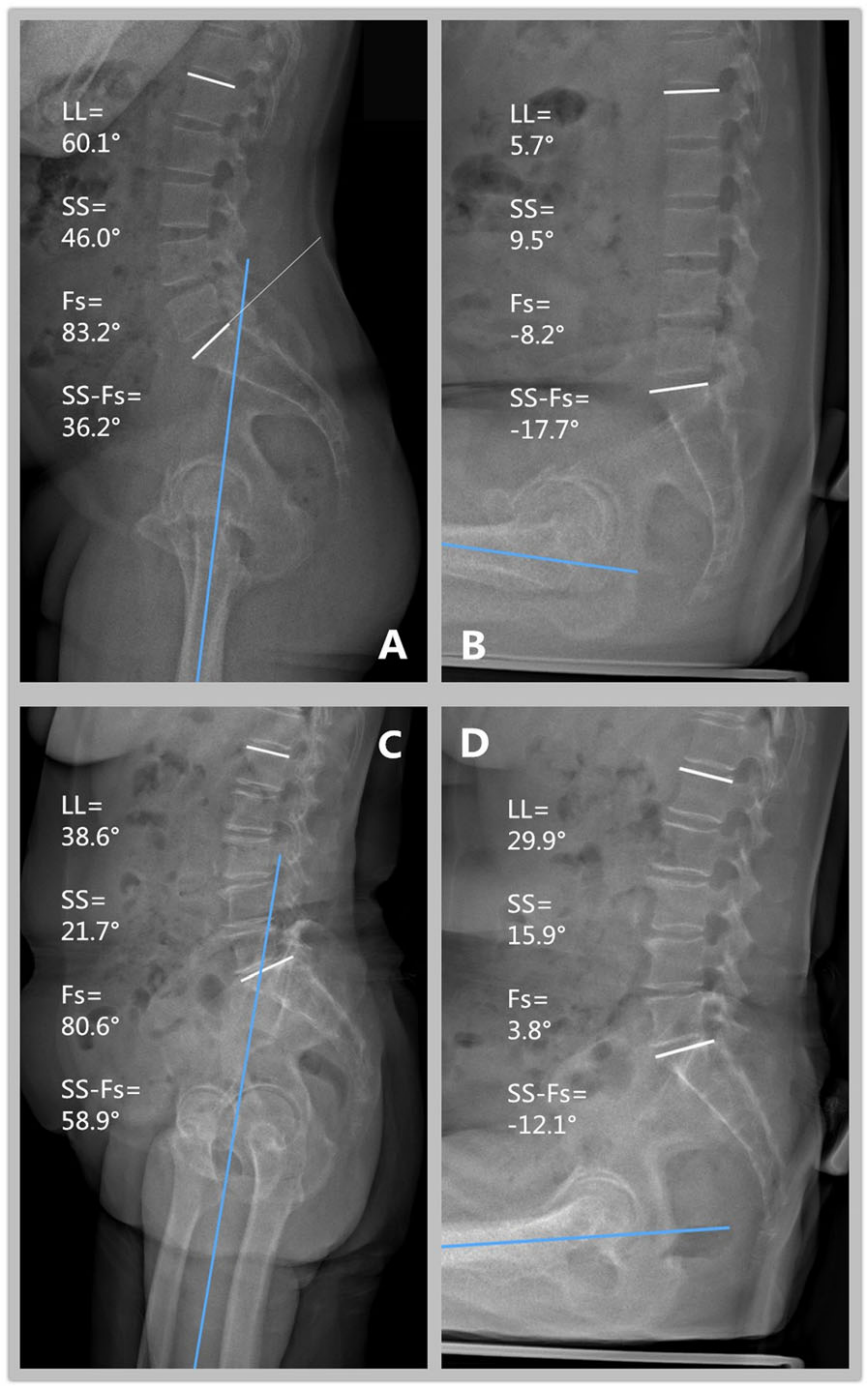
Measurement of LL, SS, Fs, and SS-Fs for patient in standing and sitting position. Patients in the control group (upper A and B) had 54.4° (change in LL from 60.1° to 5.7°) lumbar spine flexion and 53.9° (change in SS-Fs from 36.2° to − 17.7°) hip flexion from standing (A) to sitting (B). The pelvis rotated 36.5° (change in SS from 46.0° to 9.5°). Patients in the LDD group (Lower, C and D) demonstrated 8.7° (38.6° to 29.9°) lumbar spine flexion and 71° (58.9° to − 12.1°) hip flexion, from standing (C) to sitting (D). The pelvis rotated 5.8° (21.7° to 15.9°)

On changing position from standing to sitting, the femur rotated from a vertical to a horizontal position. The femur rotated 86.2° on an average; no significant difference was observed between the groups. The mean change in PI was minimal (1°), indicating a constant anatomical parameter that was not influenced by position. At the distal end of the lumbar spine, no significant difference was observed in the SS between the groups during standing. In contrast, at the proximal end of the lumbar spine, the L1 slope change was 5.9° lesser in the LDD group. In the LDD group, total spinofemoral flexion was 7° lesser and the lumbar spine flexion (LL change) was 16° lesser, and hip flexion was 7° more than that in the control group. The LDD group had an 8° smaller change in the SS and a 9° greater change in the PT (Fig. 2). The spine/hip ratio was significantly lower in the LDD group (0.3) than in the control group (0.7; *p* < 0.001; Table 2).

**Table 2:** Anatomic and positional parameters

Parameters	Control (*n* = 84) Mean ± SD	LDD (*n* = 54) Mean ± SD	*p* value	Total (*n* = 138) Mean ± SD
PI standing(°)	43.7 ± 10.3	46.9 ± 11.4	0.08	45.0 ± 10.8
PI change(°)	−1.4 ± 3.2	−1.0 ± 3.5	0.44	−1.3 ± 3.3
PT standing (°)	4.7 ± 8.0	9.2 ± 9.0	0.003	6.5 ± 8.7
PT change(°)	32.5 ± 15.3	23.4 ± 14.2	0.001	28.9 ± 15.7
SVA standing (mm)	22.1 ± 31.9	51.1 ± 51.0	< 0.001	33.5 ± 42.7
LL standing (°)	51.0 ± 9.7	46.3 ± 11.9	0.01	49.2 ± 10.8
LL change (Lumbar spine flexion) (°)	36.5 ± 15.6	20.6 ± 13.1	< 0.001	30.3 ± 16.6
SS standing(°)	38.6 ± 8.7	38.1 ± 9.0	0.78	38.4 ± 8.8
SS change(°)	30.5 ± 15.5	22.6 ± 14.2	0.03	27.4 ± 15.4
L1 slope standing(°)	12.9 ± 6.0	10.7 ± 6.7	0.04	12.1 ± 6.4
L1 slope change(°)	7.0 ± 9.0	1.1 ± 7.4	< 0.001	4.7 ± 8.9
Femur slope standing(°)	87.3 ± 4.2	85.7 ± 5.4	0.06	86.7 ± 4.8
Femur slope change(°)	86.6 ± 5.6	85.5 ± 6.5	0.30	86.2 ± 6.0
Hip flexion(°)	55.4 ± 19.2	62.3 ± 17.4	0.03	58.1 ± 18.8
Total spinofemoral flexion(°)	93.7 ± 10.4	86.5 ± 10.3	< 0.001	90.9 ± 10.9
Spine/Hip ratio	0.7 ± 0.6	0.3 ± 0.3	< 0.001	0.6 ± 0.5

*LDD* Lumbar degenerative disc disease, *PI* Pelvic incidence, *PT* Pelvic tilt, *SS* sacral slope, *SVA* Sagittal vertical axis, *LL* Lumbar lordosis.

Regression analyses were performed to investigate the relationships between the spine/hip ratio and other variables. LDD group and increasing age, and not sex, height, or weight, were significant univariate predictors of a decreased spine/hip ratio (LDD group: *p* < 0.001; age: *p* = 0.048).

## Discussion

The human body maintains the sagittal balance of the spinopelvic complex by virtue of its bony morphology and soft tissue tension. When changing position from standing to sitting, the femur rotates from a vertical to a horizontal position, which is approximately 90° in relation to the ground. The upper body is maintained upright. During this movement, the pelvis lies on both hip joints and rotates on the co-axis of the femoral heads, distributing femur movement to the trunk and over the hip joint and spine [5–8, 15, 16, 20–22]. Hip arthroplasty surgeons must identify the spinopelvic movement patterns in their patients, as patients demonstrating greater hip flexion during daily activity may be at greater risk of anterior impingement and posterior dislocation [15]. Our study assessed how the lumbar spine, pelvis, and hip joint move during change from a standing to a sitting position and the influence of lumbar disc degeneration on such spinofemoral movement.

We show that patients with LDD had 5° lesser LL on standing, indicating a relatively kyphotic posture. Blizzard et al. reported that spinal deformities decreased lumbar lordosis [16]. Patients with LDD may be unable to stand erect and may walk with the trunk leaning forwards [23, 24]. Previously, Liang et al. [25] reported that patients with lumbar disc herniation, experiencing sagittal imbalance, had a significantly increased SVA of 11.6 cm, on average. With an increased SVA and decreased LL, patients in the LDD group posteriorly rotated their pelvis by 5° more (PT) in compensation.

When changing from a standing to sitting position, patients were instructed to sit on a standard chair to achieve a horizontally placed femur. Images were acquired using the EOS system to ensure reliability. The pelvis acts as a link between the upper body and lower limbs. In 1992, Duval-Beaupere et al. introduced the concept of PI as a cornerstone for describing spinofemoral relationships [26]. The PI was not significantly different between the groups in our study and did not change from the standing to the sitting position, highlighting its anatomical characteristics.

During sitting, the L1 slope change was relatively small (4.7°). As the kyphotic thoracic spine forms a cage in combination with the ribs and respiratory muscles, a minimal sagittal ROM (0.1°) is present, as reported by Ochi et al. [8] This cage provides a stable base for the cervical spine and head. Below L1, the lumbar spine, pelvis, and femur rotate in a chain-like manner to distribute the flexion of the spinofemoral movement [21]. Spinofemoral flexion is the combination of intrinsic motion of the acetabular-femur joint and extrinsic lumbar spine movements. The total lumbar spinofemoral motion for all patients in our study was 90.9° on an average. For all the patients, the mean lumbar spine flexion was 30.3° and hip flexion was 58.1°. The hip joints bear only-two thirds of spinofemoral movement from standing to sitting. The pelvis rotated 27.4° usually posteriorly (change in SS) in all the patients. Many studies have demonstrated a SS change of 22°–27° from standing to sitting [6–8, 15, 20]. It has been reported that every 1° increase in the pelvis posterior tilt increases the acetabular component anteversion by 0.7° [22, 27–30]. The functional acetabular anteversion increased by 19° (27.4° × 0.7) on average in our patients. The pelvis movement helped to prevent posterior dislocation via a combination of reduced hip movement and greater anteversion change.

Lumbar spine degeneration alters spinopelvic alignment and motion. In our study, patients in the LDD group had 7° lesser total spinofemoral flexion. This was mainly due to decreased movement in the lumbar spine (16°). To compensate for reduced lumbar motion, patients had to recruit greater hip flexion (7°) to place the femur flat when sitting. The spine/hip ratio was significantly lower in the LDD than in the control group (0.3 versus 0.7; *p* < 0.001). The LDD group had 8° lesser pelvis rotation, which would decrease anteversion changes by 29.6% (8°/27°).

There were more female and elderly patients in the LDD group, and the mean age of patients in this group was higher than in the control group. Multivariate analysis revealed that sex was not a significant predictor of a decreased spine/hip ratio. Moreover, the cadaver and clinical studies have not indicated a significant effect of sex on lumbar disc degeneration [31, 32]. Older patients exhibit more lumbar degenerative changes, including hypertrophy of facets, degeneration of intervertebral disks, and osteophytosis of vertebrae. These phenomena would lead to disc space narrowing, loss of LL, and decreased lumbar spinal ROM. A study of 214 male patients by Burton et al. [32] revealed that reduced lumbar flexibility was multifactorial, and included lumbar disc degeneration and increased age. Furthermore, Schepper et al. reported a correlation between increased frequency of radiographic disc degeneration and age [33]. Arthroplasty surgeons should be aware that older patients with degeneration of multiple lumbar discs have a significantly different lumbar spinopelvic motion pattern.

When patients have a limited ROM in the lumbar spine, the risk of dislocation and need for revision after THA increases markedly./ Based on a 12-month follow-up, Perfetti et al. reported that, compared to the controls, the THA patients with prior spinal fusion were 7 times more likely to dislocate their prostheses (*p* < 0.01) and 4 times more likely to need revisions (p < 0.01), [10]. Sing et al. reported that the dislocation rate was 2.4% for THA patients without prior spinal fusion, 4.3% for patients with 1 to 2 levels fused, and 7.5% for patients with 3 to 5 levels fused [11]. In this study, patients with previous spine surgery were excluded. Patients with multiple degenerative lumbar discs could be more difficult to identify than those with a clear history of spine fusion surgery. Surgeons should pay particular attention to these patients with poor spinopelvic mobility, as they have greater hip flexion, increasing their risk of impingement and posterior dislocation. As both patients and surgeons are increasingly more prone to relax hip precautions postoperatively, our study outcomes may help surgeons to identify the THA candidates with stiff lumbar spine movements preoperatively. However, methods to identify these patients more easily than by X-ray imaging while standing and sitting need further investigation. Surgeons may have to place the prosthesis in a more individualized position during surgery; this has gained increased attention. It is necessary to preparing special implants, such as a dural-mobility cup, in advance, particularly when patients have additional risk factors for dislocation. Personized postoperative rehabilitation protocols should be prescribed in these cases.

This study had several limitations. First, static standing and sitting images do not fully represent a patient’s pelvic orientation during activities of daily living. Patients usually had their hip dislocated posteriorly in a specific position involving flexion, adduction, and internal rotation. Further research is required to evaluate this issue. Dislocation on sitting was rare, unless the hip was highly unstable. We did not perform assessments in a squatting position as painful hip joints hindered patients from squatting fully prior to surgery. We have been following-up these patients to record any postoperative dislocation and to relate our measurements to clinical outcomes. Second, patients preparing for THA may have stiff and painful hip motion, leading to increased lumbar spine flexion on sitting. There was no difference in the AVN stage between the two groups, and the majority of the patients were of FICAT Stage III AVN; this helped mitigate this bias. Third, the quantitative influence of the severity of lumbar disc degeneration on spinofemoral movement was not included. More patients and longer follow-up are required to analyze these differences.

## Conclusions

In conclusion, on standing, patients with LDD exhibited greater forward trunk leaning than those without LDD and had a larger compensatory pelvis posterior tilt angle. On sitting, the LDD group had reduced lumbar spine/hip flexion ratio, demonstrating greater hip joint flexion in compensation for reduced lumbar spine flexion. Surgeons should take note that older patients with LDD affecting multiple discs are at greater risk of anterior impingement and posterior dislocation after THA. Preoperative identification of such patients, intraoperative surgical technique modification, and individualized rehabilitation protocols are necessary for improving outcomes.

## Data Availability

The datasets used and/or analyzed during the current study are available from the corresponding author on reasonable request.

## Abbreviations

AP: Anteroposterior
AVN: Avascular necrosis
AVNFH: Avascular necrosis of the femoral head
Fs: Femoral slope
L1 s: L1 slope
LDD: Lumbar degenerative disc disease
LL: Lumbar lordosis angle
PI: Pelvic incidence
PT: Pelvic tilt
ROM: Range of motion
SS: Sacral slope
SVA: Sagittal vertical axis
THA: Total hip arthroplasty
UCLA: University of California at Los Angeles
